# Foraminoplasty at the Tip or Base of the Superior Articular Process for Lateral Recess Stenosis in Percutaneous Endoscopic Lumbar Discectomy: A Multicenter, Retrospective, Controlled Study with 2-Year Follow-Up

**DOI:** 10.1155/2018/7692794

**Published:** 2018-12-19

**Authors:** Jun-Song Yang, Lei Chu, Chien-Min Chen, Xiang-Fu Wang, Pei-Gen Xie, Rui Deng, Ke-Xiao Yu, Lei Shi, Zhen-Xing Zhang, Li-Min Rong, Ding-Jun Hao, Zhong-Liang Deng

**Affiliations:** ^1^Department of Spine Surgery, Honghui Hospital, Xi'an Jiaotong University, No. 76 Nanguo Road, Xi'an, Shaanxi, China; ^2^Department of Orthopaedics, The Second Affiliated Hospital, Chongqing Medical University, No. 76 Linjiang Road, District Yuzhong, Chongqing, China; ^3^Department of Neurosurgery, Changhua Christian Hospital, Changhua City, Taiwan; ^4^School of Medicine, Kaohsiung Medical University, Kaohsiung, Taiwan; ^5^College of Nursing and Health Sciences, Dayeh University, China; ^6^Department of Spinal Minimally Invasive Surgery, Gansu Provincial Hospital of Traditional Chinese Medicine, No. 418 Guazhou Road, District Qilihe, Lanzhou, Gansu, China; ^7^Department of Spine Surgery, Third Affiliated Hospital, Sun Yat-sen University, Guangzhou, China

## Abstract

**Objective:**

To compare the clinical efficacy and complications which obtained foraminoplasty at the tip or base of the superior articular process (SAP) for the patients with lateral recess stenosis treated by percutaneous endoscopic lumbar discectomy (PELD).

**Methods:**

Between January 2015 and January 2016, 156 patients of lumbar disc herniation accompanying with lateral recess stenosis were treated with PELD in five tertiary hospitals and fulfilled the 2-year follow-up. Among them, 78 patients obtained a foraminoplasty at the tip of SAP (group A), and foraminoplasty at the base of SAP was performed in the other 78 cases (group B). Clinical efficacy was evaluated using the visual analog scale (VAS) score for back and leg pain, Oswestry Disability Index (ODI), and 36-item Short-Form Health Survey (SF-36) score. The intervals of follow-up were scheduled at 1 month, 3 months, 6 months, 1 year, and 2 years after surgery.

**Results:**

Mean operative duration is shorter in group B (55 versus 61 min, P = 0.047). Only one case belonged to group A could not tolerate the neural irritation and required conversion to an open procedure. During the surgery, no dura tears, cauda equina syndrome, or infections were observed. 5 patients experienced transient dysesthesia located at the exiting nerve in group A, while no cases complained dysesthesia in group B. 2 cases who suffered temporary motor weakness all belonged to group A. A total of 5 cases obtained a revision surgery after recurrence in the follow-up, in which 3 patients belonged to group A. Compared to the preoperative data, significant improvements in VAS scores of low back pain and sciatica, ODI, and SF-36 PCS and MC were observed in the follow-up, respectively (P < 0.05, respectively). However, no statistical difference was observed at all time-points after surgery between these two groups (P > 0.05, respectively).

**Conclusions:**

For the patients of LDH accompanying with lateral recess stenosis, compared with the routine foraminoplasty at the tip of SAP, our modified foraminoplastic technique does not only change place of foraminoplasty to the base of SAP but also simplified puncture process in transforaminal PELD. Although there was no significant difference in symptom relief, the modified foraminoplasty showed the advantages in decreasing the incidence of postoperative neural dysfunction and reducing operation time.

## 1. Introduction

Since the introduction of the percutaneous discectomy by Kambin in 1973 [1], transforaminal percutaneous endoscopic lumbar discectomy (PELD) has recently been an increasingly popular surgical procedure to treat lumbar disc herniation, which bridges the gap between conservative treatment and traditional surgery. Numerous studies have proved that PELD provide successful outcomes comparable to conventional open or microendoscopic surgery  [2–7]. However, it showed the advantages in controlling muscular trauma, shortening hospital stay, and maintaining the spinal segment stability [2, 7, 8]. As the neural decompression was performed under a single port, how to precisely establish an ideal working cannula toward the targeted lesion is the base of PELD. Superior articular process (SAP) is the main culprit between the posterolateral rod-shaped endoscope and the anteromedial dura sac, especially for the elderly patients with hypertrophic facet joint and lateral recess stenosis. To address the problem, some authors have raised the technique of endoscopic foraminoplasty by using a reamer, drill, or laser, which widens the lumbar intervertebral foramen and facilitates the establishment of working sheath [9–15]. However, foraminoplasty-related complications, like postoperative dysesthesia and motor weakness associated with the nerve root injury, are the principal concerns of performing foraminoplasty in PELD [9, 15]. The classical foraminoplasty, the so-called Tessys technique described by Schubert and Hoogland, was toward to the tip of SAP [16, 17]. As the SAP, the posterior border of intervertebral lumbar foramen, without the protection from the outside sheath, the trephine makes direct contact with paraforamen soft tissue, causing concerns about damage to the exiting nerve root and dura sac; thus extent of foraminoplasty, the extent of SAP removing, was limited [18]. Anatomatically, because the base of SAP was far away from the exiting nerve root, a foraminoplasty which targeted the base of SAP could provide thorough foraminoplasty decreasing the iatrogenic injury of nerve root. To compare the clinical efficacy and complications which obtained foraminoplasty at the tip or base of the SAP, we first perform a multicenter study for the patients with lateral recess stenosis treated by PELD.

## 2. Method

Between January 2015 and January 2016, 156 patients of lumbar disc herniation accompanying with lateral recess stenosis were treated with PELD in five tertiary hospitals and fulfilled the 2-year follow-up. Among them, the first 78 patients obtained a foraminoplasty at the tip of SAP (group A), and foraminoplasty at the base of SAP was performed in the secondary 78 cases (group B). Patient demographics and characteristics are summarized in Table 1. Approval to conduct the study was granted by the ethics committees of hospitals. Institutional Review Board approved informed consent and protocols were also provided to all patients.

Inclusion criteria are as follows: (1) clinical signs of neurological deficit including radiculopathy, paresthesia, and motor weakness; (2) symptoms corresponding with preoperative magnetic resonance imaging (MRI) of computed tomography (CT) scan and concordant with lateral recess stenosis (the anteroposterior diameter of the lateral recess was less than 4 mm); (3) unsatisfactory conservative treatment for at least 6 weeks; (4) patients who wrote informed consent to participate in this evaluation and further follow-ups.

Exclusion criteria are as follows: (1) definite segmental instability (the anterior or posterior displacement > 3 mm or the angle change of the endplate > 15 degrees on the dynamic radiography); (2) cauda equina syndrome with severe central canal stenosis (less than 10 mm) on preoperative MRI or CT; (3) highly migrated nucleus pulposus beyond the low rims of adjacent pedicles; (4) high iliac crest without enough space for addressing the disc herniation at the L5-S1 level via a posteriolateral transforaminal approach; (5) suspected infection or Malignant diseases.

## 3. Surgical Technique

**Figure 1:**
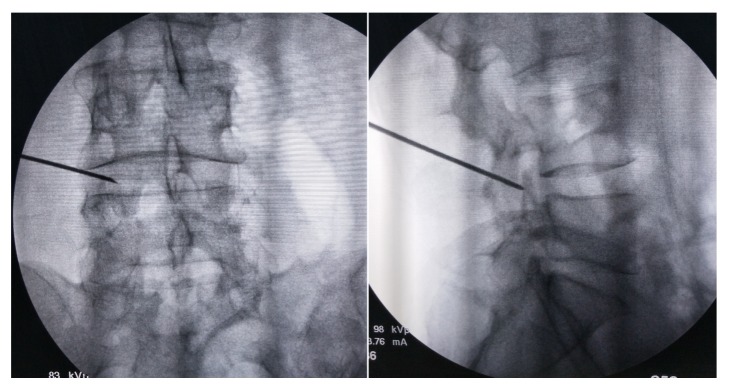
The puncture needle was first placed on the dorsal surface of facet joint, which was confirmed by the anteroposterior (left) and lateral (right) views of fluoroscopy.

**Figure 2:**
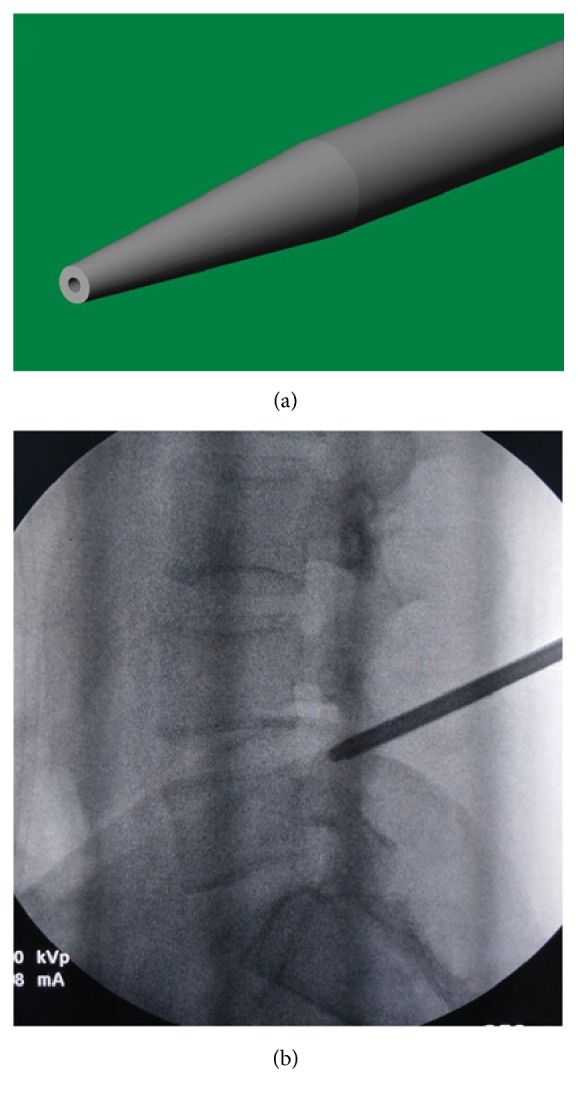
The guide rod is blunt with a pencil head-shaped end (Panel (a)); it was placed on the dorsal surface of facet joint, which was confirmed by the lateral (Panel (b)) view of fluoroscopy.

**Figure 3:**
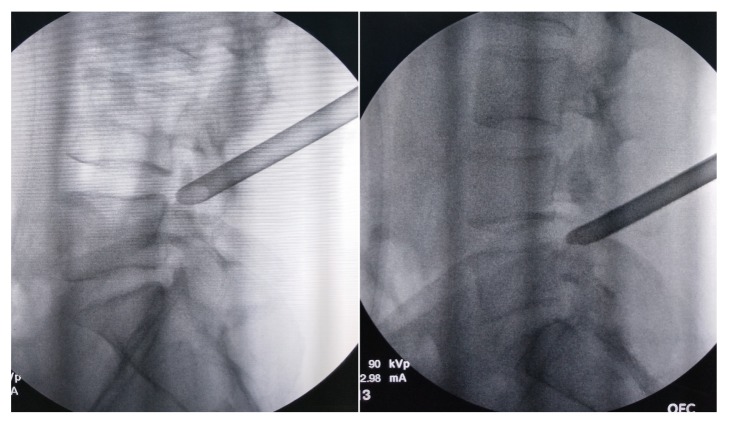
The base (left) or tip (right) of the ventral part of SAP was covered by the protective cannula at the lateral views of fluoroscopy.

**Figure 4:**
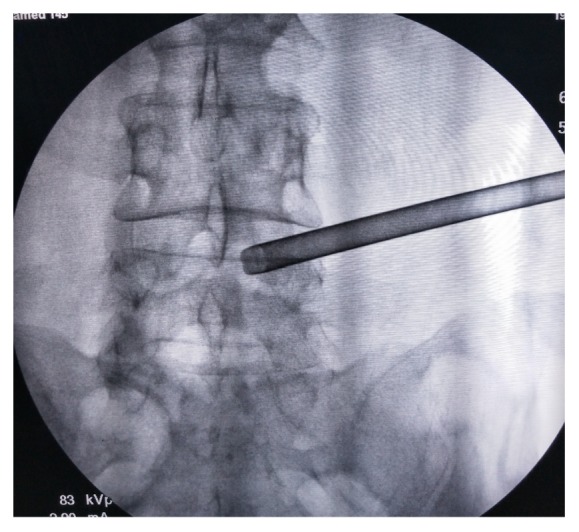
Because the ventral hypertrophic SAP was removed as much as possible, the protective cannula can be placed beyond the medial margin of inferior articular process, which was confirmed by the anteroposterior view of fluoroscopy.

All patients obtained PELD via a transforaminal approach under local anesthesia in the prone position. Dexmedetomidine hydrochloride (0.5 *μ*g/kg bolus, followed by 0.1–0.5 *μ*g/kg/hour) was injected intravenously could improve the patient's surgical tolerance. Entry point of needle was determined by the point of intersection between the horizontal line and the oblique caudal directional line tangent with the tip of SAP. A 16G spinal needle was utilized in the puncture process, whose diameter was larger than the routine 20G spinal needle and better to adjust the puncture trajectory in the strong back muscles. For avoiding the iatrogenic injury to the peritoneal sac, the puncture trajectory was slightly dorsal toward rather the intervertebral foramen. The spinal needle was first placed on the dorsal surface of facet joint (Figure 1). Under the guidance of spinal needle, a topical anesthesia was performed in the capsule of facet joint. Another spinal needle was introduced to perform infiltrating anesthesia into the paraspinal muscles with 8–10 ml of 0.5% lidocaine from the skin at the insertion site and along the needle entry tract. Then, the stylet of spinal needle was replaced by a guide wire, and the outside sheath was removed. A stab incision of approximately 5 mm long was made around the guide wire. Along with the guide wire, a blunt guide rod with a pencil head-shaped end was introduced and placed at the surface of SAP (Figure 2), and the inner guide wire was retrieved. Under the guidance of lateral bony margin of SAP, the tapered end of the cannulated guide rod was slid into the intervertebral foramen and fixed by the around bony fracture and soft tissue. According to the foraminoplasty toward to the tip or base of the SAP, the guide rod was placed at the upper or lower part of intervertebral foramen. The protective cannula could be introduced toward the SAP along the guide rod. A topical anesthesia could be added according the tolerance of patients thorough the protective cannula. As the distal end of protective cannula was e bevel half shaped, which could cover the tip or base of the ventral part of SAP (Figure 3). A trephine was inserted into the protective cannula to perform foraminoplasty via the transforaminal approach. As the tip of protective cannula anchored in foramen is like a fulcrum, the lateral side of trephine together with the protective cannula was downward nearly horizontally to remove the ventral hypertrophic SAP as much as possible, even the inferior articular process of cranial vertebra, and expand the narrow lateral recess (Figure 4). It was notable that the process of trephine advancing should be close monitored under fluoroscopy. Protective cannula was replaced with working cannula. An endoscope (SPINENDOS GmbH, Munich, Germany) with a working channel of 4.3-mm outside diameter is introduced. Because the narrow lateral recess and intervertebral foramen were adequately enlarged, additional maneuvers like levering the cannula to make it more horizontal could be easily achieved without the irritation to the ventral exiting nerve root. After removing the hypertrophic ligamentum flavum, the nerve root and the protruded nucleus pulposus were gradually recognized under endoscopic visualization. When the protruded nucleus pulposus was thoroughly removed, the crevasse of annulus fibrosus was detected. Annuloplasty was performed to prevent the recurrent herniation of intradiscal nucleus pulposus. The surgery was halted when the satisfactory decompression of traversing nerve root and dura sac was confirmed.

## 4. Postoperative Management and Outcome Assessment

**Figure 5:**
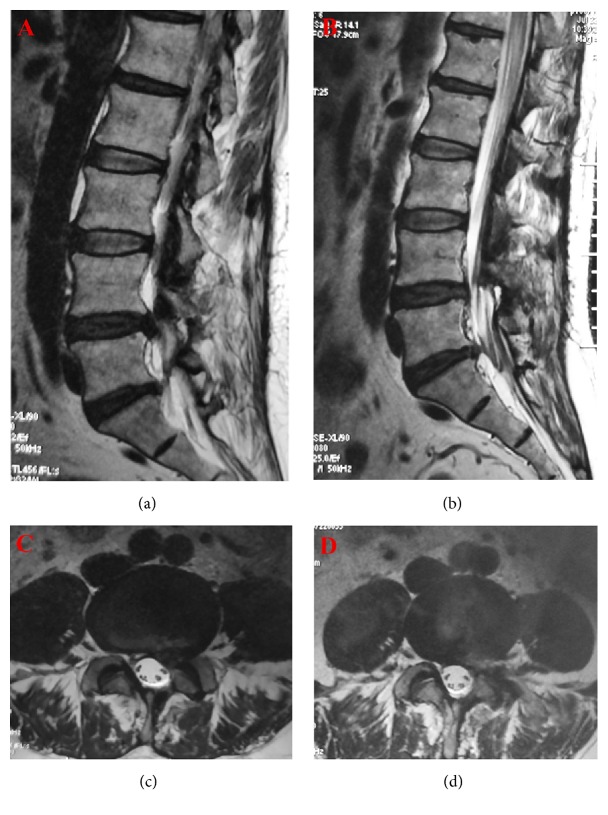
In the preoperative MRI, the sagittal (Panel (a)) and axial (Panel (b)) planes of T2-weighted imaging showed a lumbar disc herniation at the level L4/5. The decompression was satisfactory, which was confirmed at the sagittal (Panel (c)) and axial (Panel (d)) planes of the postoperative MRI.

**Figure 6:**
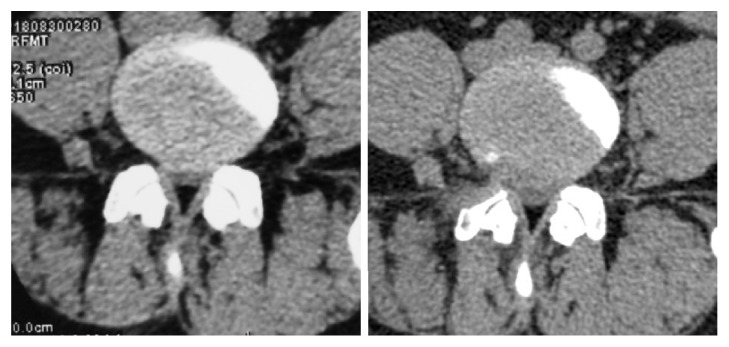
Compared to the preoperative CT scan (Panel A), the herniated disc to the right side was totally remove at the postoperative CT scan (Panel B). Notably, the hypertrophic SAP at the right side was partially removed.

To decrease the possibility of recurrence of postoperative disc herniation, the lumbar brace was recommend to wear for approximately 4 weeks to ensure that the ruptured annular fibrosis could achieve satisfactory healing. The intensity of back and leg pain was assessed by visual analog scale (VAS) score retrospectively. Functional outcomes were assessed by using Oswestry Disability Index (ODI) score and SF-36. The intervals of follow-up were scheduled at 1 month, 3 months, 6 months, 1 year, and 2 years after surgery. The physical examinations and clinical scores were performed by another surgeon who did not participate in surgery procedures. The related complications, including postoperative dysesthesia and motor weakness, were also recorded. Postoperative MRI and CT examinations were obtained in all patients routinely at postoperative 1 day to detect whether residual disc was occurred (Figures 5 and 6). Dynamic lumbar radiography was recommended at the final follow-up.

## 5. Statistical Analysis

Statistical analyses were performed with SPSS 11.5 software (SPSS Inc., Chicago, IL). Preoperative and postoperative VAS scores of back and leg pain as well as ODI and SF-36 values were analyzed with ANOVA retrospectively. P < 0.05 was considered as significant.

## 6. Results

Mean operative duration is shorter in group B (55 versus 61 min, P = 0.047). Only one case in the group A could not tolerate the neural irritation and required conversion to an open procedure. During the surgery, no dura tears, cauda equina syndrome, or infections were observed in the present case series. 5 patients experienced transient dysesthesia located at the exiting nerve in the group A, while no cases complained dysesthesia in group B. 2 cases suffered temporary motor weakness all belonged to group A. A total of 5 cases obtained a revision surgery after recurrence in the follow-up, in which 3 patients belonged to group A. Preoperative and postoperative VAS scores of low back pain and sciatica, ODI, and SF-36 PCS and MC are summarized in Tables 2–6, respectively. Compared to the preoperative data, a significant improvement in VAS scores of low back pain and sciatica, ODI, and SF-36 PCS and MC were observed in the follow-up, respectively (P < 0.05, respectively). However, no statistical difference was observed at all time-points after surgery between these two groups (P > 0.05, respectively).

## 7. Discussion

As the most widely used endoscopic approach in the treatment of LDH, transforaminal PELD to optimize the route to the spinal canal percutaneously has been described since the late 1990s and obtained satisfactory clinical outcome [19]. However, among the perioperative complications, incomplete or unsuccessful removal of disc fragments is relatively often and clinically worrisome. Unsatisfactory establishment of working channel and residual disc are the main reason of failure of PELD. For complex lumbar disc herniation (LDH), such as central, migrated, and axillary type, and the failure rate of PELD without foraminoplasty is as high as 4.3%-10.3% [20, 21]. As the intervertebral foramen at the lower lumbar region is gradually decreased, the SAP could block the surgical field to the anterior epidural space and limit the manipulated space to place the working channel through the intervertebral foramen access the lesions. In the LDH patients accompanying with lateral recess stenosis, how to widen the intervertebral foramen and lateral recess is quite vital in the process of PELD. Foraminoplasty could provide direct visualization of the anterior epidural space via thorough decompression at the ventral and dorsal structure, especially widening of the foramen by undercutting of ventral part of the SAP and removing the foraminal ligament [22].

The described instrumentations utilized for foraminoplasty include endoscopic burr, side-firing laser, reamers and trephine, etc [9, 15, 16, 21–25]. Endoscopic burr and side-firing laser could remove the SAP under endoscopic visualization improving surgical safety. However, these tools are so tiny to affect the efficiency of foraminoplasty, in which lateral recess was not enough to enlarge because of the restriction of the working channel of the rigid endoscope. Additionally, the process of undercutting of SAP while using a high-speed endoscopic burr or side-firing laser may potentially could lead to vibration stimulation or thermal damage, inducing iatrogenic injury to exit nerve root [24, 25]. Knight et al. observed that temporary nerve irritation noted in 19% of patients postoperatively when a side-firing laser was used for performing foraminoplasty under PELD [9], while Ahn et al. reported the rate of postoperative dysesthesia is 6.1% after endoscopic foraminoplasty with an endoscopic high-speed drill [15]. Compared with endoscopic burr and side-firing laser, a trephine or bone reamer is an economical and time-saving equipment to undercut the hypertrophic SAP or osteophyte under fluoroscopic guidance. However, without the monitor under continuous visualization endoscopic and protection of outside sheath, foraminoplasty with trephine or reamer carries the risk of injury to the exiting and traversing nerve root, which may produce leg pain and neurological dysfunction in the affected extremity. Li et al. modified the current technique of foraminoplasty, in which they change the place of foraminoplasty from the tip of SAP to the base of SAP [26, 27]. As the place of foraminoplasty is far away from the exiting nerve root, the incidence of postoperative nerve root dysfunction is theoretically low. Additionally, a protective cannula was introduced into the process of foraminoplasty to act as a barrier between exiting nerve root and the removing bony structure of SAP. The design of duck-mouth-like distal end can facilitate the protective cannula covering the cambered SAP. When levering the cannula to make it more horizontal, downward or upward tilting, the foraminoplasty could accomplish in individual trajectory under the protection of outer cannula. Besides the tip of SAP, the horizontal part of the SAP and lateral recess medial to the pedicle are the key points of foraminoplasty. However, a foraminoplasty with larger area could not only affect the exit nerve root, but also put the transversing nerve root at risk. As the medial part of SAP is covered by the capsule of facet joint and the ligamentum flavum, they are a buffer to prevent the undercutted portion of the SAP migrated into the spinal canal compressing the transversing nerve root and dura sac. As the bone of the horizontal part of the SAP and lateral recess medial to the pedicle is quite thick and hard [28], an uncontrolled reaming with a greater depth could beyond the limitation of covering ligament structure of SAP. Thus, the trephine could penetrate the medial wall of SAP and violate the transversing nerve root and dura sac. To controlled depth of foraminoplasty, we designed a spacer, which can be fixed at the caudal side of trephine to limit the depth, because the foraminoplasty is not directly toward the exit nerve root but focuses on the surround bony structure. With the process of foraminoplasty, the intervertebral foramen and the lateral recess are gradually enlarged. The created area is a buffer space between the working sheath and the exiting nerve root to facilitate inserting the working cannula more deep toward the targeted lesion decreasing the risk of the iatrogenic injury to the exiting nerve root. That could explain why the incidence postoperative neural dysfunction is low in the group A.

We have improved the puncture procedure and simplified the process. There is no need to place the puncture needle exactly to the tip or the base of SAP but only need to place the puncture needle at the lateral side of SAP. Once the puncture needle is attached to the surface of the facet joint, a blunted guide rod with larger diameter than the puncture needle was introduced along with the guide wire. The guide rod could slide along with the lateral and ventral surface of articular joint and into the intervertebral foramen. Thus, the puncture procedure is simplified; the radiation of intraoperative fluoroscopy and the operation time is controlled. The unique design that the guide rod is blunted with a pencil head-shaped end could facilitate the guide rod sliding and anchoring into the intervertebral foramen. Because the trajectory was slightly toward the dorsal side rather the ventral side and the blunted guide rod replaced with the sharp puncture needle, the risk of perforation of abdominal visceral organs in the puncture process is low.

Excessive removal of the facet joints has been proved to be associated with spinal instability after open surgery [29, 30]. As excessive bone was also removed in the foraminoplasty, whether it could influence the stability of the lumbar segment is not widely explored. Osman et al. made the first cadaveric study to explore the pathological anatomy, intervertebral foraminal area, and flexibility changes between posterior and transforaminal decompression [31]. A 45.5% increase in the intervertebral foraminal area was possible; there was no flexibility change, and minimal anatomic damage to the spine was noted after transforaminal decompression. Li et al. [26, 27] consider that when the anteromedial third of the superior facet, the anterior part of inferior facet, and the portion of the joint between them were removed, there was no violation of the anatomic integrity of the lumbar spine in the procedure of foraminoplasty; the risk of surgically induced instability was minimized after PELD. However, the postoperative stability was not radiologically evaluated. In this controlled study, there is no postoperative instability observed in the surgical spinal unit in the 2-year follow-up. We believe that, besides preserving the anatomic integrity of the lumbar spine, a nearly complete reservation of ligamental and muscular structure is beneficial for maintaining the spinal stability.

Different from the previous studies [26, 27], we used a local anesthetic agent accompanied with dexmedetomidine. Dexmedetomidine hydrochloride is a potent and highly selective alpha-2 agonist, which has been safely used for various diagnostic and therapeutic procedures to facilitate patient comfort [32]. In chronic subdural hematoma evacuation, Surve et al. have proven that dexmedetomidine sedation with local anesthesia was a safe and effective technique for patients undergoing a burr hole procedure [33]. In our previous study, we have successfully removed the epidural leaked cement under local anesthesia accompanied with dexmedetomidine sedation [34].

## 8. Conclusion

For the patients of LDH accompanying with lateral recess stenosis, compared with the routine foraminoplasty at the tip of SAP, our modified foraminoplastic technique is not only changed place of foraminoplasty to the base of SAP but also simplified puncture process in transforaminal PELD. Although there was no significant difference in symptom relief, the modified foraminoplasty showed the advantages in decreasing the incidence of postoperative neural dysfunction and reducing operation time.

## Figures and Tables

**Table 1:** Summary of demographic and treatment level.

Baseline characteristic	Group A	Group B
Female gender (%)	43 (44.9)	49 (37.2)
Mean age (yrs) (range)	54.3 (45-65)	53.5 (52-68)
Treatment level		
L3-4 (%)	12 (15.4)	15 (19.2)
L4-5 (%)	43 (55.1)	41 (52.6)
L5-S1 (%)	23 (29.5)	22 (28.2)

SAP: superior articular process.

**Table 2:** Changes of preoperative and postoperative VAS scores of low back pain (*x* ± *s*).

**Time point **	**Pre-operation **	**1 months postoperatively**	**3 months postoperatively**	**6 months postoperatively**	**1 year postoperatively**	**2 years postoperatively**
Group A	5.1±0.7	3.2±0.6	2.5±0.8	2.1±0.4	1.6±0.3	1.5±0.4

Group B	5.0±0.9	3.0±0.7	2.8±0.6	2.0±0.3	1.7±0.5	1.4±0.5

*VAS: visual analogue scale.*

**Table 3:** Changes of preoperative and postoperative VAS scores of sciatica (*x* ± *s*).

**Time point **	**Pre-operation **	**1 months postoperatively**	**3 months postoperatively**	**6 months postoperatively**	**1 year postoperatively**	**2 years postoperatively**
Group A	7.1±0.8	2.2±0.8	2.0±0.5	1.8±0.5	1.6±0.3	1.5±0.3

Group B	7.0±0.9	2.0±0.7	1.9±0.4	1.7±0.4	1.5±0.4	1.4±0.2

*VAS:  visual analogue scale.*

**Table 4:** Changes of preoperative and postoperative ODI scores (*x* ± *s*).

**Time point **	**Pre-operation**	**1 months postoperatively**	**3 months postoperatively**	**6 months postoperatively**	**1 year postoperatively**	**2 years postoperatively**
Group A	50.1±6.9	34.5±5.6	20.4±5.3	18.1±4.3	16.8±3.8	14.6±3.2

Group B	50.4±5.3	33.8±5.4	20.9±4.4	17.8±4.5	16.9±3.1	14.7±3.0

*ODI:  Oswestry Disability Index.*

**Table 5:** Changes of preoperative and postoperative SF-36 MC scores (*x* ± *s*).

**Time point**	**Pre-operation**	**1 year postoperatively**	**2 years postoperatively**
Group A	28.4±8.1	50.8±9.3	65.2±8.1

Group B	29.1±7.7	51.3±10.1	64.1±7.3

*MC:* ***mental component.***

**Table 6:** Changes of preoperative and postoperative SF-36 MC scores (*x* ± *s*).

**Time point**	**Pre-operation**	**1 year postoperatively**	**2 years postoperatively**
Group A	28.4±8.1	55.8±9.3	62.2±8.1

Group B	29.1±7.7	56.3±10.1	61.1±7.3

*MC:* ***mental component.***

## Data Availability

The research related data used to support the findings of this study are restricted by the Ethics Committee of Honghui Hospital, Xi'an Jiaotong University, in order to protect patient privacy. Data are available from Zhong-Liang Deng, email address: 215069125@qq.com, for researchers who meet the criteria for access to confidential data
